# Patterns of engagement in a digital mental health service during COVID-19: a cohort study for children and young people

**DOI:** 10.3389/fpsyt.2023.1143272

**Published:** 2023-07-27

**Authors:** Aynsley Bernard, Santiago de Ossorno Garcia, Louisa Salhi, Ann John, Marcos DelPozo-Banos

**Affiliations:** ^1^Kooth Digital Health, London, United Kingdom; ^2^Swansea University Medical School, Swansea, United Kingdom

**Keywords:** COVID-19, pandemic, engagement, digital mental health, mental health, children and young people, machine learning, clustering

## Abstract

**Introduction:**

The COVID-19 pandemic increased public use of digital mental health technologies. However, little is known about changes in user engagement with these platforms during the pandemic. This study aims to assess engagement changes with a digital mental healthcare service during COVID-19.

**Methods:**

A cohort study based on routinely collected service usage data from a digital mental health support service in the United Kingdom. Returning users aged 14–25 years that interacted for a maximum of two months were included. The study population was divided into pre-COVID and COVID cohorts. Demographic and usage information between cohorts were compared and usage clusters were identified within each cohort. Differences were tested using Chi-squared, two-sample Kolmogorov–Smirnov tests and logit regressions.

**Results:**

Of the 624,103 users who joined the service between May 1, 2019, and October 1, 2021, 18,889 (32.81%) met the inclusion criteria: 5,048 in the pre-COVID cohort and 13,841 in the COVID cohort. The COVID cohort wrote more journals; maintained the same focus on messaging practitioners, posting discussions, commenting on posts, and having booked chats; and engaged less in writing journals, setting personal goals, posting articles, and having *ad-hoc* chats. Four usage profiles were identified in both cohorts: one relatively disengaged, one focused on contacting practitioners through chats/messages, and two broadly interested in writing discussions and comments within the digital community. Despite their broad similarities, usage patterns also exhibited differences between cohorts. For example, all four clusters had over 70% of users attempting to have *ad-hoc* chats with practitioners in the pre-COVID cohort, compared to one out of four clusters in the COVID cohort. Overall, engagement change patterns during the COVID-19 pandemic were not equal across clusters. Sensitivity analysis revealed varying strength of these defined clusters.

**Discussion:**

Our study identified changes in user activity and engagement behavior within a digital mental healthcare service during the COVID-19 pandemic. These findings suggest that usage patterns within digital mental health services may be susceptible to change in response to external events such as a pandemic. Continuous monitoring of engagement patterns is important for informed design and personalized interventions.

## Introduction

Research suggests that the COVID-19 pandemic has exacerbated the mental health crisis across many countries, including the United Kingdom (UK) (1). Furthermore, there is a growing body of evidence highlighting effects of the pandemic and consequent lockdowns on children and young people’s (CYP) mental health (2–4). At the same time, contacts and interactions with all types of healthcare services reduced dramatically during the pandemic (5). This was also true for mental health-related contacts, particularly face-to-face contacts, with patients having to access to video and over-the-phone contacts (6, 7). Electronic mental healthcare and telemedicine rapidly became the “new normal” (8). By mid-2020, more than 80% of high-income countries shifted to digital mental health technologies to replace or supplement in-person mental health consultations (1).

The use of digital mental health technologies has provided data for several machine learning studies focusing on patterns of engagement within user-led digital support systems. These studies have illustrated several different ways in which mental health support can be personalized in a digital setting: based on engagement type, frequency of access, session duration, timing, and clinical outcomes (9–11). Segmenting users according to behaviors within digital mental health services can be a first step in personalizing support and improving design effectiveness (12).

However, to the best of our knowledge, there are no studies exploring whether user engagement and behaviors within these platforms are subject to change during major events like the COVID-19 pandemic, in which patterns of engagement can be disrupted and present some challenges to machine learning assumptions or solutions based on this type of information. At the same time, such knowledge could inform the use of digital mental health interventions (an important psychological support component during the COVID-19 pandemic) in future disasters (13) and help to resource and prevent overload or saturation of healthcare provision using data-driven technology and decisions.

We hypothesize that stable engagement types exist within Kooth, which could inform the personalization of services. The COVID-19 pandemic provides a unique opportunity to test this hypothesis, as it was an exceptional situation that could potentially affect user engagement patterns with digital mental health services. This cohort study aims to assess changes in engagement within a digital mental health service in the UK during the COVID-19 pandemic, comparing routinely collected usage data between a pre-COVID and a COVID cohort of users.

## Materials and methods

We used data from Kooth Digital Health,^1^ the UK’s largest provider to the National Health Service of web-based online mental health support (14). This service provides mental health support and interventions through its pseudonymous platform to CYP aged 11–25 years at no cost to the service users. Users can self-refer and find out about Kooth from school, online promotion, primary and secondary health services, social media or word of mouth.

The service allows CYP to self-direct their experience, interacting with their preferred type of support from a range of service features: personal journals, goal setting, discussion boards, articles, asynchronous therapeutic messaging, and live text-based counseling. Comprehensive safeguarding procedures are adhered to by moderators and practitioners following user interactions with the service. Demographic and usage information is stored across databases that can be linked at an individual level under the legal basis of ‘legitimate interest’ as it informs service improvements (15). In this study, data was used from 1 May 2019 to 31 December 2021.

### Study sample

This study relied on data from 1 May 2019 to 31 December 2021. We included users between the ages of 14–25 years, and who had consented to have their non-identifiable demographic and service usage information used for research purposes. Users who were flagged by practitioners as not having Gillick competence (16) were excluded from the analysis.

To ensure sufficient journey and engagement information per service user, only returning users (i.e., with two or more log-ins) were included in the analysis. Users with a journey longer than 56 days were excluded from the dataset to avoid outliers, in that 99.03% of returning users aged 14–25 had a usage period of 56 days or less. To reduce bias from cut-off or cohort-crossing usage periods, users were excluded if their registration was within 56 days of the end date for each cohort dataset.

We divided users into two cohorts: pre-COVID and COVID. The World Health Organization declared COVID-19 a global pandemic on 11 March 2020 (17). Hence, we defined pre-COVID and COVID cohorts as users who signed up from 1 May 2019 to 11 January 2020 (256 days), and from 11 March 2020 to 1 October 2021 (570 days), respectively.

### Measures

Demographic variables of interest collected routinely included ethnicity (‘Asian’, ‘Black’, ‘Mixed’, ‘White’ and ‘Other’), gender (‘Female’, ‘Male’ and ‘Non-binary’) and age group (‘14–17’ and ‘18–25’) at the time of registration. We measured interaction with the service through a number of service usage variables: 3 continuous variables (‘usage period’, ‘engagement’ and ‘activeness’) recording the overall level of interaction; and 8 dichotomous variables recording whether users made use of each component of the service (e.g., journals, discussions, *ad-hoc* chats). Table 1 provides the complete list of variables, including activity type, with details.

**Table 1:** Characteristic, usage and experience variables of interest.

Variable Type	Variable	Description
Characteristic		
	Signup Age	Measured in years and split into two groups: 14–18 and 18–25 years.
	Ethnicity	One of ‘Asian’, ‘Black’, ‘Mixed’, ‘White’ and ‘Other’. If a user has ‘Other’ as their ethnicity status, this could be because they selected ‘Other’ or because they did not state their ethnicity.
	Gender	One of ‘Agender’, ‘Female’, ‘Gender Fluid’ and ‘Male’. ‘Agender’ and ‘Gender Fluid’ are grouped into ‘Non-binary’ due to low counts.
Usage		
Engagement Metrics	Usage Period	Days between first and last login (absolute).
	Engagement	Number of active days* divided by Usage Period (defined above).
	Activeness	Number of activities divided by active days.*
Self Help	Journal Entry	Text journal entry and emoji submitted by a user to signify how the user feels.
	Personal Goal Created	Goal set by a user for themselves.
Community Engagement	Article Created	Article submitted by a user.
	Discussion Created	Discussion thread started by a user.
	Comment Created	Comment added to an article or discussion by a user.
Asynchronous Practitioner Engagement	Message Sent	Message sent to a practitioner by a user.
Synchronous Practitioner Engagement	Drop-in Chat Requested	Impromptu chat requested by joining the chat queue.
	Booked Chat Requested	Booked chat with a practitioner scheduled.
Experience		
Asynchronous Practitioner Engagement	Administrative message received	Administrative message sent from practitioner to user.
	Therapeutic message received	Therapeutic message sent from practitioner to user that includes an assessment, is consistent with a model of intervention and is intended to change behavior.
Synchronous Practitioner Engagement	Successful chat	User and practitioner are in an *ad-hoc* chat for >5 min.
	Failed chat	User and practitioner do not successfully stay within an *ad-hoc* chat for >5 min.

*‘Active days’ is the number of days a user has interacted with the service.

### Analysis

We performed two-stepped analyses: (1) comparison of pre-COVID and COVID cohorts and (2) identification of usage profiles within pre-COVID and COVID cohorts. All data processing and analyses were done in python v3.9.2 (18). Packages sshtunnel 0.4.0, psycopg2-binary 2.9.5, pymysql 1.0.2, and python-bigquery 3.3.5 (19–22) were used to query data sources and construct the measures. We used scipy 1.9.3 (23) and statsmodels 0.13.2 packages (24) for statistical modeling and tests. The threshold for statistical significance for all value of *p*s was set at *p* < 0.05.

The main analyses were preceded by an assessment of the generalizability of our results for the service population, comparing separately for pre-COVID and COVID cohorts the study sample (returning, research consenting Kooth users aged 14–25 with a journey of ≤56 days) with the corresponding wider study population (all returning Kooth users aged 14–25). We used the Mann–Whitney U test (25) to measure differences in signup age as a continuous variable, and the Chi-squared test (26) to measure differences in ethnicity and gender.

In the first step of the analysis, we measured proportion and 95% confidence intervals (CI) estimated by Wilson score with continuity correction (27). We measured variations between the pre-COVID and COVID cohorts using Chi-squared tests (26) for demographic variables; two-sample Kolmogorov–Smirnov tests (28) for continuous usage variables; and logit regressions for dichotomous usage variables (as outcomes) using ‘*cohort*’ (i.e., pre-COVID or COVID) as the response variable and controlling for demographic and service change covariates.

In the second step, user groups were identified separately for pre-COVID and COVID cohorts through clustering of the usage variables. Prior to clustering, continuous usage variables were transformed to a logarithmic scale to limit the negative impact of large outliers (29). The usage data was then transformed into a binary indicator of whether the user had interacted with each component of the service, to address the sparsity of the data and improve the efficacy of dimensionality reduction (30). We applied Multiple Correspondence Analysis to reduce dimensionality and allow for the use of Euclidean-based clustering (31).

We ran a sensitivity analysis in which we applied KMeans, Birch, DBSCAN, and Gaussian Mixture Models on the resulting dataset to explore differences across clustering algorithms. We computed the Silhouette Coefficient as (*b_i_ – a_i_*)*/max*(*a_i_, b_i_*), with *a_i_* the mean intra-cluster distance of sample *i*, *b_i_* the mean nearest-cluster distance of sample *i*, and *N* the number of samples. This ranges from −1 (worst) to 1 (best) – values near 0 indicate overlapping clusters (32) (Supplementary Tables S3, S4). The cluster number choice was made by inspecting the silhouette analysis plots for the highest scoring algorithms: Birch and KMeans for 2 to 5 clusters (Supplementary Figures S1–S4). After assessing these plots and clusters, we decided to present the output of Birch in the main text, but we ran the full analysis using KMeans for comparison.

We made a deliberate decision not to include service user demographics in the clustering algorithm to minimize the potential biasing effect of demographic factors on the identification of engagement behavior patterns. By doing so, we ensured that the resulting clusters were based solely on the observed engagement behaviors and not influenced by demographic characteristics. Instead, we were interested in observing whether different engagement profiles naturally had demographic differences.

Clusters were then further explored using Chi-squared tests (26) for demographic variables (not used to compute the clusters), Anderson-Darling tests (33) for k-samples for continuous usage variables; and logit regressions for dichotomous usage variables (as outcomes) with ‘*cluster*’ as the response and adjusting for covariates age group, ethnicity and gender (an overall *value of p* for variable ‘*cluster*’ was calculated through a log-likelihood test). For each cluster, we show proportion, 95% CIs and calculated value of *ps* per variable.

## Results

### Study sample

**Figure 1:**
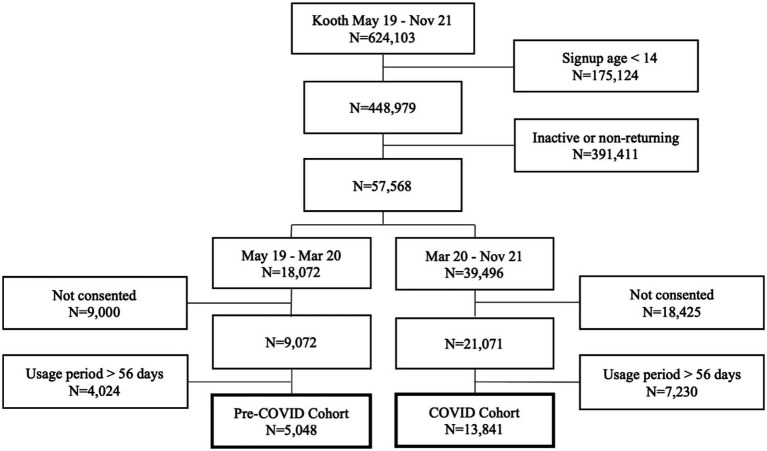
Study cohort flow diagram.

Of the 624,103 individuals who joined Kooth between May 1, 2019, and October 1, 2021, 57,568 (12.82%) were returning users aged 14–25 years. Of these, 18,889 (32.81%) met all the inclusion criteria: 5,048 in the pre-COVID cohort and 13,841 in the COVID cohort. The number of signups per day increased from 19.72 in the pre-COVID cohort to 24.28 in the COVID cohort. The most common place for service users to find out about Kooth is school, which remained consistent across both cohorts but with altered proportions (pre-COVID: 45.38%, COVID: 34.24%). Full details of the cohort selection procedure are in Figure 1.

When comparing demographic distributions between the pre-COVID and COVID cohorts and their corresponding population of interest, we found no significant difference in signup age (*p* = 0.220) and ethnicity (*p* = 0.999) for the pre-COVID cohort, and a significant difference for the COVID cohort (*p* < 0.001) with mean signup age of 0.78 years older and a 0.47 percentage points increase in user proportion that did not select an ethnicity option. For both cohorts, we found no significant differences in gender (*p* > 0.888).

### Comparison between pre-COVID and COVID study cohorts

Full results of the comparison between pre-COVID and COVID cohorts are in Table 2.

**Table 2:** Variable distributions for pre-COVID and COVID cohorts.

Variable	Pre-COVID	COVID	Adjusted *p* value
Summary statistics			
Signups	5,048	13,841	
Signups per day	19.72	24.28	
Demographic variables/Control variables not used for clustering			
Gender			<0.001
Female	3,899 (77.24% [76.06, 78.37])	10,406 (75.18% [74.46, 75.89])	
Male	971 (19.24% [18.17, 20.35])	2,629 (18.99% [18.35, 19.66])	
Non-binary	178 (3.53% [3.05, 4.07])	806 (5.82% [5.45, 6.23])	
Age group			<0.001
14–17	4,683 (92.77% [92.02, 93.45])	12,290 (88.79% [88.26, 89.31])	
18–25	365 (7.23% [6.55, 7.98])	1,551 (11.21% [10.69, 11.74])	
Ethnicity group			<0.001
White	4,259 (84.37% [83.34, 85.35])	11,280 (81.5% [80.84, 82.14])	
Asian	331 (6.56% [5.91, 7.27])	917 (6.63% [6.22, 7.05])	
Black	141 (2.79% [2.37, 3.28])	471 (3.4% [3.11, 3.72])	
Mixed	239 (4.73% [4.18, 5.36])	727 (5.25% [4.89, 5.64])	
Other	78 (1.55% [1.24, 1.92])	446 (3.22% [2.94, 3.53])	
White	4,259 (84.37% [83.34, 85.35])	11,280 (81.5% [80.84, 82.14])	
Service usage variables/Dependent variables used for clustering			
Period*		-	0.016
Engagement*		-	0.074
Activeness*		-	<0.001
Journal entry	3,716 (73.61% [72.38, 74.81])	11,675 (84.35% [83.74, 84.95])	<0.001
Personal goal created	1,007 (19.95% [18.87, 21.07])	2,418 (17.47% [16.85, 18.11])	<0.001
Article created	296 (5.86% [5.25, 6.55])	466 (3.37% [3.08, 3.68])	<0.001
Discussion created	1,051 (20.82% [19.72, 21.96])	2,741 (19.8% [19.15, 20.48])	0.251
Comment created	1,644 (32.57% [31.29, 33.87])	4,772 (34.48% [33.69, 35.27])	0.008
Message sent	771 (15.27% [14.31, 16.29])	2,237 (16.16% [15.56, 16.78])	0.129
*Ad-hoc* chat	3,791 (75.1% [73.89, 76.27])	7,816 (56.47% [55.64, 57.29])	<0.001
Booked chat	196 (3.88% [3.38, 4.45])	403 (2.91% [2.64, 3.21])	0.001
Service experience variables/Observational variables not used for clustering			
Administrative message received	475 (9.41% [8.63, 10.25])	7,553 (54.57% [53.74, 55.4])	<0.0001
Therapeutic message received	680 (13.47% [12.56, 14.44])	7,152 (51.67% [50.84, 52.5])	<0.0001
Successful chat	1,567 (31.04% [29.78, 32.33])	2,611 (18.86% [18.22, 19.52])	<0.0001
Failed chat	1,065 (21.1% [19.99, 22.24])	2,675 (19.33% [18.68, 19.99])	0.008

Reported values are absolute counts, percentages with 95% confidence intervals, and adjusted *p* values assessing whether variables changed during COVID compared to pre-COVID.

**Figure 2:**
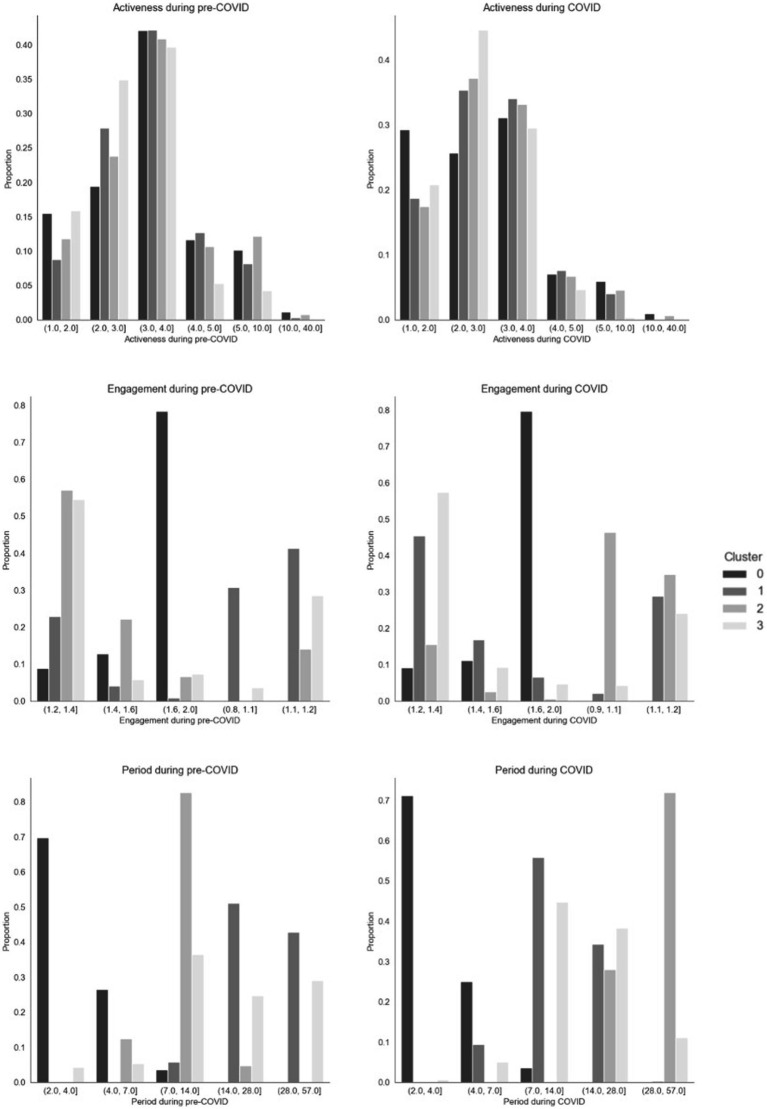
Distribution plots for period, activeness and engagement variables across pre-COVID and COVID Birch clusters.

*Continuous variable. See Figure 2.

Signup age, and the proportion of users who reported gender as ‘Non-binary’ increased during the pandemic, as did the proportion of users reporting ‘Black’, ‘Mixed’ or ‘Other’ ethnicity against a decrease in those reporting ‘White’. More users had relatively longer usage periods during the pandemic, with similar engagement rates but a more active interaction with the service. The COVID cohort wrote more journals; maintained the same focus on messaging practitioners, posting discussions, commenting on posts, and having booked chats; and engaged less in writing journals, setting personal goals, posting articles, and having *ad-hoc* chats. A visual representation of similarities and differences in service usage between pre-COVID and COVID periods can be seen in the right-hand side of Supplementary Figures S1–S4.

### Identification of usage clusters

During optimization of the clustering algorithms, the silhouette score varied between 0.14 and 0.46 for the pre-COVID cohort and between 0.16 and 0.43 for the COVID cohort across 24 hyperparameter configurations. Full results can be seen in Supplementary Tables S1 and S2 for the pre-COVID and COVID cohorts, respectively. There was no clear best configuration for both pre-COVID and COVID cohorts. Inspecting both the results of the silhouette analysis (Supplementary Figures S1–S4) and the obtained clusters, we decided to continue with the output of 4 usage clusters for each cohort.

### Comparison between pre-COVID and COVID usage clusters

Figure 2 and Tables 3–6 show the size, characteristics, and usage profiles for each cluster. We observed some broad similarities between pre-COVID and COVID usage clusters. In both cases, cluster sizes were highly unbalanced, with the largest and smallest clusters containing 53.80 and 3.74% of pre-COVID users, respectively, and 51.29 and 2.15% of COVID users, respectively. The largest cluster in both cohorts was also the one with the shortest enrolment period (1–2 days) and the least engaged in practitioner-based interventions. Similarly, the smallest cluster in both cohorts was also the one with highest proportion of females, older users, and most engaged with practitioner-based interventions. The remaining two clusters where the most interested in community-based interventions.

**Table 3:** Control variables across Birch engagement clusters for the pre-COVID cohort.

Variable	C0	C1	C2	C3
Pre-COVID Birch clusters				
Summary statistics				
Signups	2,716	1,345	798	189
Proportion of all pre-COVID signups	53.80%	26.64%	15.81%	3.74%
Signups per day	10.61	5.25	3.12	0.74
Control variables not used for clustering				
Gender (*p* = 0.684)				
Female	2091 (76.99% [75.37, 78.53])	1,045 (77.7% [75.39, 79.84])	612 (76.69% [73.63, 79.49])	481 (80.84% [77.48, 83.8])
Male	535 (19.7% [18.25, 21.24])	245 (18.22% [16.24, 20.37])	158 (19.8% [17.18, 22.71])	91 (15.29% [12.63, 18.41])
Non-binary	90 (3.31% [2.7, 4.06])	55 (4.09% [3.16, 5.28])	28 (3.51% [2.44, 5.02])	23 (3.87% [2.59, 5.73])
Age group (*p* = 0.052)				
14–17	2,520 (92.78% [91.75, 93.7])	1,233 (91.67% [90.07, 93.03])	757 (94.86% [93.1, 96.19])	173 (91.53% [86.69, 94.72])
18–25	196 (7.22% [6.3, 8.25])	112 (8.33% [6.97, 9.93])	41 (5.14% [3.81, 6.9])	16 (8.47% [5.28, 13.31])
Ethnicity group (*p* = 0.01)				
Asian	183 (6.74% [5.85, 7.74])	101 (7.51% [6.22, 9.04])	32 (4.01% [2.85, 5.61])	15 (7.94% [4.87, 12.68])
Black	75 (2.76% [2.21, 3.45])	37 (2.75% [2.0, 3.77])	26 (3.26% [2.23, 4.73])	3 (1.59% [0.54, 4.56])
Mixed	138 (5.08% [4.32, 5.97])	54 (4.01% [3.09, 5.2])	34 (4.26% [3.06, 5.89])	13 (6.88% [4.06, 11.41])
Other	36 (1.33% [0.96, 1.83])	29 (2.16% [1.51, 3.08])	10 (1.25% [0.68, 2.29])	3 (1.59% [0.54, 4.56])
White	2,284 (84.09% [82.67, 85.42])	1,124 (83.57% [81.49, 85.45])	696 (87.22% [84.72, 89.36])	155 (82.01% [75.91, 86.83])
Messages received				
Message received	447 (16.46% [15.11, 17.9])	344 (25.58% [23.32, 27.98])	147 (18.42% [15.88, 21.26])	88 (46.56% [39.59, 53.67])

Reported values are absolute counts and percentages with 95% confidence intervals. Chi-squared tests across clusters returned *p* < 0.001 for all variables except otherwise specified.

**Table 4:** Usage variables across Birch engagement clusters for the pre-COVID cohort.

Variable	C0	C1	C2	C3
Pre-COVID Birch clusters				
Signups	2,716	1,345	798	189
Dependent variables used for clustering				
Journal entry	2078 (76.51% [74.88, 78.07])	992 (73.75% [71.34, 76.04])	552 (69.17% [65.88, 72.28])	94 (49.74% [42.68, 56.8])
Personal goal created	520 (19.15% [17.71, 20.67])	310 (23.05% [20.88, 25.37])	153 (19.17% [16.59, 22.05])	24 (12.7% [8.68, 18.2])
Article created	154 (5.67% [4.86, 6.6])	104 (7.73% [6.42, 9.28])	32 (4.01% [2.85, 5.61])	6 (3.17% [1.46, 6.75])
Discussion created	512 (18.85% [17.42, 20.37])	326 (24.24% [22.02, 26.6])	194 (24.31% [21.46, 27.41])	19 (10.05% [6.53, 15.17])
Comment created	810 (29.82% [28.13, 31.57])	504 (37.47% [34.92, 40.09])	287 (35.96% [32.71, 39.35])	43 (22.75% [17.35, 29.24])
Message sent	344 (12.67% [11.47, 13.97])	251 (18.66% [16.67, 20.83])	131 (16.42% [14.01, 19.15])	45 (23.81% [18.3, 30.37])
*Ad-hoc* chat	1914 (70.47% [68.73, 72.16])	1,065 (79.18% [76.93, 81.27])	625 (78.32% [75.33, 81.04])	187 (98.94% [96.22, 99.71])
Booked chat (no value of *p*)	0 (0.0% [0.0, 0.14])	6 (0.45% [0.2, 0.97])	1 (0.13% [0.02, 0.71])	189 (100.0% [98.01, 100.0])
Observational variables not used for clustering				
Successful chat	581 (21.39% [19.89, 22.97])	529 (39.33% [36.75, 41.97])	271 (33.96% [30.76, 37.32])	186 (98.41% [95.44, 99.46])
Failed chat	425 (15.65% [14.33, 17.06])	319 (23.72% [21.52, 26.06])	169 (21.18% [18.48, 24.15])	152 (80.42% [74.18, 85.45])

Reported values are absolute counts and percentages with 95% confidence intervals. Logistic regression models adjusted for demographic variables returned *p* < 0.001 for all variables unless otherwise specified. Some results are omitted due to convergence issues. Cases where ‘no value of *p*’ is stated indicate where the algorithm did not converge.

**Table 5:** Control variables across Birch engagement clusters for the COVID cohort.

Variable	C0	C1	C2	C3
COVID Birch clusters				
Summary statistics				
Signups	7,099	3,847	2,597	298
Proportion of all COVID signups	51.29%	27.79%	18.76%	2.15%
Signups per day	12.45	6.75	4.56	0.52
Control variables not used for clustering				
Gender				
Female	5,219 (73.52% [72.48, 74.53])	2,925 (76.03% [74.66, 77.36])	2026 (78.01% [76.38, 79.56])	236 (79.19% [74.23, 83.42])
Male	1,451 (20.44% [19.52, 21.39])	704 (18.3% [17.11, 19.55])	420 (16.17% [14.81, 17.64])	54 (18.12% [14.16, 22.89])
Non-binary	429 (6.04% [5.51, 6.62])	218 (5.67% [4.98, 6.44])	151 (5.81% [4.98, 6.78])	8 (2.68% [1.37, 5.21])
Age group (*p* = 0.109)				
14–17	6,311 (88.9% [88.15, 89.61])	3,413 (88.72% [87.68, 89.68])	2,316 (89.18% [87.93, 90.32])	250 (83.89% [79.29, 87.63])
18–25	788 (11.1% [10.39, 11.85])	434 (11.28% [10.32, 12.32])	281 (10.82% [9.68, 12.07])	48 (16.11% [12.37, 20.71])
Ethnicity group (*p* = 0.016)				
Asian	432 (6.09% [5.55, 6.67])	282 (7.33% [6.55, 8.2])	190 (7.32% [6.38, 8.38])	13 (4.36% [2.57, 7.32])
Black	255 (3.59% [3.18, 4.05])	125 (3.25% [2.73, 3.86])	81 (3.12% [2.52, 3.86])	10 (3.36% [1.83, 6.07])
Mixed	367 (5.17% [4.68, 5.71])	197 (5.12% [4.47, 5.86])	150 (5.78% [4.94, 6.74])	13 (4.36% [2.57, 7.32])
Other	246 (3.47% [3.06, 3.92])	125 (3.25% [2.73, 3.86])	65 (2.5% [1.97, 3.18])	10 (3.36% [1.83, 6.07])
White	5,799 (81.69% [80.77, 82.57])	3,118 (81.05% [79.78, 82.26])	2,111 (81.29% [79.74, 82.74])	252 (84.56% [80.02, 88.22])
Messages received				
Message received	5,217 (73.49% [72.45, 74.5])	3,022 (78.55% [77.23, 79.82])	2,147 (82.67% [81.17, 84.08])	273 (91.61% [87.91, 94.25])

Reported values are absolute counts and percentages with 95% confidence intervals. Chi-squared tests across clusters returned *p* < 0.001 for all variables except otherwise specified.

**Table 6:** Usage variables across Birch engagement clusters for the COVID cohort.

Variable	C0	C1	C2	C3
COVID Birch clusters				
Signups	7,099	3,847	2,597	298
Dependent variables used for clustering				
Journal entry	6,147 (86.59% [85.78, 87.36])	3,141 (81.65% [80.39, 82.84])	2,237 (86.14% [84.76, 87.41])	150 (50.34% [44.69, 55.97])
Personal goal created	1,170 (16.48% [15.64, 17.36])	642 (16.69% [15.54, 17.9])	585 (22.53% [20.96, 24.17])	21 (7.05% [4.66, 10.53])
Article created	184 (2.59% [2.25, 2.99])	177 (4.6% [3.98, 5.31])	100 (3.85% [3.18, 4.66])	5 (1.68% [0.72, 3.87])
Discussion created	1,186 (16.71% [15.86, 17.59])	916 (23.81% [22.49, 25.18])	623 (23.99% [22.39, 25.67])	16 (5.37% [3.33, 8.54])
Comment created	2091 (29.45% [28.41, 30.53])	1,626 (42.27% [40.71, 43.83])	991 (38.16% [36.31, 40.04])	64 (21.48% [17.19, 26.49])
Message sent	831 (11.71% [10.98, 12.47])	692 (17.99% [16.81, 19.23])	647 (24.91% [23.29, 26.61])	67 (22.48% [18.11, 27.56])
*Ad-hoc* chat	3,529 (49.71% [48.55, 50.87])	2,331 (60.59% [59.04, 62.13])	1,697 (65.34% [63.49, 67.15])	259 (86.91% [82.61, 90.28])
Booked chat (no value of *p*)	1 (0.01% [0.0, 0.08])	3 (0.08% [0.03, 0.23])	101 (3.89% [3.21, 4.7])	298 (100.0% [98.73, 100.0])
Observational variables not used for clustering				
Successful chat	794 (11.18% [10.47, 11.94])	831 (21.6% [20.33, 22.93])	717 (27.61% [25.92, 29.36])	269 (90.27% [86.37, 93.14])
Failed chat	1,066 (15.02% [14.2, 15.87])	769 (19.99% [18.76, 21.28])	610 (23.49% [21.9, 25.16])	230 (77.18% [72.09, 81.58])

Reported values are absolute counts and percentages with 95% confidence intervals. Logistic regression models adjusted for demographic variables returned *p* < 0.001 for all variables unless otherwise specified. Cases where ‘no value of *p*’ is stated indicate where the algorithm did not converge.

At the same time, there were substantial differences between the pre-COVID and COVID clusters. For example, gender differences were only significant in the COVID cohort. The unengaged cluster (C0) was less active during the pandemic. Over 70% of users in all pre-COVID clusters requested and *ad-hoc* chat with a practitioner, compared to only one COVID cluster. The two clusters interested in community-based interventions (C1 and C2) showed opposite trends in usage period and engagement. One of these (C2) was also the least engaged on creating articles before COVID, but the second most engaged during COVID. The cluster engaging the most with practitioner-based intervention had the highest proportion of users who selected Asian and Mixed ethnicity pre-COVID, and the lowest during COVID.

Our sensitivity analysis had mixed results (Supplementary Figure S5; Supplementary Tables S3–S5). In the pre-COVID period, the obtained clusters were substantially different to those of Birch, although we still found a cluster of disengaged used (this time even more disengaged). Of the remaining clusters, one was focused on both self-help and community-based interventions, and the other two focused on practitioner-based interventions and had moderate interest in community-based interventions. The size of pre-COVID clusters based on KMeans was also much more valanced, with each accounting for 20–30% of users. Meanwhile, KMeans’ result during COVID was similar to Birch’s, with a disengaged cluster, a cluster focused on practitioner-based interventions, and a cluster focused on community-based interventions. The fourth cluster was also relatively focused on community-based interventions. Cluster sizes were also similar to Birch’s.

## Discussion

### Key findings

We found changes in the usage of Kooth, a UK mental health digital service, by users aged 14–25 years during the COVID-19 pandemic. While the number of signups per day increased, these users were less engaged with the service, most prominently with less activity within each log-in (albeit usage periods were longer on average) and focusing less on creating articles and discussions and requesting *ad-hoc* chats with practitioners. This excess of users during the pandemic may be driven by a lack of capacity on traditional mental health services, a desire to ‘protect’ these services, and/or fear of COVID-19 infection in physical settings. We also identified changes in the user experience, with more users being asynchronously contacted and fewer having live chats with practitioners during the pandemic. This is likely the result of service changes implemented to manage the observed increase in the demand, like practitioners actively contacting users.

We conducted cluster analyses individually in each time period (before and during the COVID-19 pandemic) and identified four clusters or usage profiles: one relatively disengaged, one focused on contacting practitioners through chats/messages, and two broadly interested in writing discussions and comments within the digital community. The disengaged profile is likely an extension of our initial observation on the high proportion of users not returning to the system after one visit, as this profile is also the largest of the four (>50% of users), highlighting the importance of this type of interaction and user preference for digital interventions. Users seeking only contact with practitioners returned to the system sporadically. This is a fitting strategy for them, since there are natural idling times between messages and chats. Users more interested on posting articles, discussions and comments seemed to be the most committed overall, with relatively longer usage periods, engagement and activeness metrics. These seemed to be the most valanced users in terms of engagement, showing also high interactions in personal- and practitioner-based interventions. All clusters had over 70% of users requesting *ad-hoc* chats with practitioners, highlighting the importance of this type of interaction for digital interventions.

Pre-COVID and COVID usage profiles, despite being grossly similar, had some stark differences particularly with the two community-focused clusters. These two clusters exhibited opposite changes on some activity in the platform (e.g., practitioner-based interventions), even swapping their ranking as most/least engaged as a result in some instances. They also swapped the length of usage period and engagement. We originally thought that these differences may have been artificially introduced by moving from three to four clusters, but inspection of the Silhouette plots (Supplementary Figures S1, S2) revealed that this step gave way to the practitioner-focused cluster (i.e., the two community-engaged clusters were already present with three clusters). We also observed differences in demographic variables not used for clustering. These may explain part of the usage profile changes between pre-COVID and COVID within the platform, but the demographic differences pertained to a small proportion of the study sample, and therefore unlikely to explain the full range of such changes. Therefore, significant external events such as pandemics may impact how users interact with digital mental health services and may affect how to effectively identify patterns of engagement to form profiles.

Our sensitivity analysis led to a similar conclusion: that usage profiles are susceptible to significant external events. However, it also showed that the resulting usage profiles are not always strongly defined in our data, and thus the selection of clustering algorithm may have a big impact on the results – this may also be weakness of our data, rather than the methodology itself. As such, the utilization of usage profiles to inform the ongoing design of these services and the recommendations of personalized interventions may not be an optimal strategy – at least not during major events and not without the right data, careful sensitivity analyses and a strong methodology leading to robust outcomes.

Our clustering analysis revealed changes in service usage not readily apparent from the analysis using the full pre-COVID and COVID cohorts. Most prominently, despite community engagement variables decreasing (articles created) or not changing (discussions and comments created) during the COVID-19 pandemic, the influx of users focused on community engagement increased. Therefore, even though community engagement decreased during the COVID-19 pandemic, 2 out of 5 users that registered during this period in fact directed their attention to community-based activity. This effect may have been driven by the lockdown, self-isolation, and social distancing measures in place during the pandemic, and thus reflect users longing for social interaction, especially in young people (34). In general terms, different clusters show different patterns of change during COVID. From a methodological point of view, these results suggest that clustering analysis may be a useful tool in the analysis of service usage and its change over time, as it can provide insight into previously hidden patterns.

### Comparison with prior research

Prior work on mental health service usage profiling incorporates time and typically tries to understand where a user is in their lifetime with a service (9–11). However, since the average user’s time with the service studied here is less than two weeks (pre-COVID: 12.35 [12.91], COVID: 12.90 [13.20]), we simplified the analysis by assuming fixed usage profiles throughout the users’ journey.

Prior work on mental health service usage profiling incorporates outcome variables and relies on a single time period for examination (9–11). They typically found between 3 and 5 usage profiles, mostly focused on the level of engagement. Since we did not have access to outcome variables in our analysis, direct comparison with other study results is not possible. However, we found a similar number of usage profiles within each cohort, some overall more engaged than others, but we also found differences in the type of engagement, as discussed above.

Previous mental health studies have shown a widespread deterioration of the population’s mental health during the COVID-19 pandemic (1), but disproportionately so for young adults and minoritized gender and ethnic groups (35, 36). We found corresponding increases in the number of signups to the service (from 19.72 users/day to 24.28 users/day). The proportion of users increased for adults, users who selected ‘Black’, ‘Mixed’ or ‘Other’ ethnicity and users who selected ‘Agender’ or ‘Gender Fluid’ gender, but not in the proportion of females compared to males.

### Strengths and limitations

We have assessed changes in the way users interact with a digital mental health service before and after the COVID-19 pandemic started in the UK, using routinely collected usage data from 18,969 users across 30 months, including the first two waves of the pandemic. We explored whether these differences varied across user types, themselves defined using clustering techniques on usage information. To the authors’ knowledge, this is the first work to study how engagement behaviors within a digital mental healthcare service change during a global crisis of this kind.

We approached the use of clustering techniques not as a central part of the research, but as a tool to answer our research question (i.e., whether usage profiles changed during the COVID-19 pandemic). As such, our methodological decisions were not driven by clustering performance, but by domain knowledge (e.g., access routes of users to the different parts of the service, and the way their interactions are recorded) to ensure the relevance of all the included variables. Additionally, we validated and compared the resulting clusters using traditional statistical methods and exploring variable distributions.

There were limitations surrounding the study population, as it included only 32.81% of the total population of users ever using the service (27.93% from the full pre-COVID cohort and 35.04% from the full COVID cohort). Nevertheless, of age, gender and ethnicity, the study sample only differed from the whole population on signup age.

The digital mental health service examined moved through several product and service improvements, potentially influencing usage. This translates to unmeasured impact of changes which prevent us from establishing with complete certainty a relationship between the changes solely attributable to COVID-19 pandemic. Other potential mental health covariates were not available, like socioeconomic status or simultaneous engagement with other services, which has been shown to change during the pandemic (36, 37). The time period of data collection is also not consistent across cohorts, so there is a possibility that changes could be due to seasonality effects or other confounders.

Engagement with digital mental health services may also be subject to variation based on service availability, making it challenging to determine which digital behaviors are genuinely influenced by the service user and not by changes in the platform and resources to provide support. We made efforts to control for changes due to service availability in terms of messages sent to service users by practitioners. However, controlling for this factor becomes exceedingly difficult in an active, naturalistic environment where resource changes can occur at different times and in various regions where the service operates.

Our study was limited to a UK-only service, which restricted our ability to compare engagement data with similar services in other countries. Therefore, the generalizability of our findings is limited to the UK context, and caution should be exercised in extrapolating our results beyond the digital service examined.

### Future research

Future research into CYP engagement would benefit from incorporating mental health measures before and after engagements. This would allow us to explore if measure responses predict engagement with digital services when combined with age, ethnicity and gender. There are known barriers in access to mental health services, and therefore understanding engagement patterns with digital mental health services can provide an early look into engagement preferences or barriers. We had data on mental health measures associated with this study, but opportunities for completion of these measures within the system were based on engagement preferences and were therefore biased. Hence, we decided to exclude outcome measures with a view to investigating outcomes in a separate study.

This study focuses only on returning users. For Kooth users aged 14–25, 87.18% of users do not return to the site after initial signup which leaves a large portion of the service user population uninvestigated. This drop-off could be due to implementation barriers such as lack of personalization or human capacity (38), and it is similar to that reported by other digital platforms (39). Future research is needed to understand the difference between returning and non-returning users, and how to maximize the potential of brief engagement vs. more continuous and regular engagement.

Our main finding, that usage profiles are affected by major events, puts into question the stability of usage profiles using clustering methods of data based on engagement. Further analysis over periods without major catastrophic events is required to ascertain whether changes in usage profiles can also occur naturally (i.e., without the influence of major events), but this also highlights the importance of examining and accounting for such events when machine learning algorithms are used for cluster and designing products and services and optimization.

This study focuses on user engagement changes between pre-COVID and COVID cohorts. Future work should investigate the long-term impact of the pandemic on mental health. It would be beneficial to explore whether mental health issues during the pandemic have regressed back to their mean or if they have persisted, as well as the role of community support in mental health during the pandemic. This may involve longitudinal studies to track changes in service engagement and mental health outcomes over time.

Kooth is a standalone digital mental health platform that provides online support for children and young people – it is not specifically designed to be integrated into face-to-face mental health care or other health care systems. While Kooth can be accessed independently by users, it may also be used as part of a blended care approach, where digital interventions are combined with traditional face-to-face services. However, the extent to which Kooth is adaptable to a blended care approach is beyond the scope of this study and warrants further investigation.

## Conclusion

The study of the effect of the COVID-19 pandemic on digital mental health services is particularly relevant, as these remained uninterrupted, while face-to-face services paused or changed provision. We explored the user activity and engagement behavior within a digital mental healthcare service and identified changes in these digital profiles during the COVID-19 pandemic. This indicates that usage profiles are not suitable to inform service design or provide personalized interventions yet, as they are susceptible to change due to events like a pandemic. However, usage profiles can provide important insight into the analysis of such changes in digital behavior and can help us better understand digital mental health service user populations and contribute to future disaster management procedures (13).

While digital mental health interventions can be powerful support tools, particularly in periods when traditional face-to-face services lack capacity or space, a better understanding of user engagement with these systems and how it changes over time is needed to fully unlock their potential, alongside other important considerations such as effectiveness, usability, and equity of access.

## Data availability statement

The data analyzed in this study is subject to the following licenses/restrictions: Under Kooth’s privacy policy, data can only be shared with trusted partners for research studies dedicated to improving the service. Requests to access these datasets should be directed to AB, abernard@kooth.com.

## Ethics statement

The studies involving human participants were reviewed and approved by Swansea University Ethics Committee. Written informed consent from the participants’ legal guardian/next of kin was not required to participate in this study in accordance with the national legislation and the institutional requirements. Study data covered only individuals that provided informed consent for their data to be utilized for research purposes.

## Author contributions

AB and MDP-B defined the original concept for the study, conducted the comparison of clusters, and wrote the original draft. AB prepared the data and conducted the cluster analysis. AB, SdOG, and MDP-B interpreted the results. All authors reviewed the original draft and contributed to the final version.

## Funding

AJ and MDP-B were funded by UKRI – Medical Research Council through the DATAMIND Hub (MRC reference: MR/W014386/1).

## Conflict of interest

AB, SdOG, and LS are employed and receive honorarium by Kooth plc. MDP-B was contracted by Kooth plc as a consultant for this work.

The remaining author declares that the research was conducted in the absence of any commercial or financial relationships that could be construed as a potential conflict of interest. The authors declare that this study received funding from Kooth Digital Health. The funder had the following involvement in the study: study design, data collection and analysis, interpretation of the results, and preparation of the manuscript.

## Publisher’s note

All claims expressed in this article are solely those of the authors and do not necessarily represent those of their affiliated organizations, or those of the publisher, the editors and the reviewers. Any product that may be evaluated in this article, or claim that may be made by its manufacturer, is not guaranteed or endorsed by the publisher.

## Footnotes

^1^ Kooth.com

## Abbreviation

CYP: children and young people
