# Supplementary material for: Patterns of engagement in a digital mental health service during COVID-19: a cohort study for children and young people
Source: Front Psychiatry. 2023 Jul 27;14:1143272. doi: 10.3389/fpsyt.2023.1143272 (PMC10415812; doi:10.3389/fpsyt.2023.1143272)
Supplement: Supplementary file 1 [file Data_Sheet_1.docx]

**Supplementary Table 1.** Clustering algorithm optimization for the pre-COVID cohort across 24 Birch hyperparameter configurations.

| **Model Type** | **Parameter Configuration** | **Clusters** | **Silhouette Score** |
| --- | --- | --- | --- |
| Birch | threshold=0.2 | 2 | 0.4572 |
| Birch | threshold=0.05 | 2 | 0.4413 |
| Birch | threshold=0.1 | 2 | 0.4346 |
| Birch | threshold=0.1 | 3 | 0.3230 |
| Birch | threshold=0.05 | 3 | 0.2996 |
| Birch | threshold=0.1 | 4 | 0.2875 |
| Birch | threshold=0.05 | 5 | 0.2776 |
| Birch | threshold=0.05 | 4 | 0.2767 |
| Birch | threshold=0.2 | 3 | 0.2582 |
| Birch | threshold=0.05 | 6 | 0.2246 |
| Birch | threshold=0.2 | 9 | 0.2025 |
| Birch | threshold=0.1 | 5 | 0.2019 |
| Birch | threshold=0.05 | 8 | 0.2009 |
| Birch | threshold=0.05 | 7 | 0.1949 |
| Birch | threshold=0.2 | 8 | 0.1915 |
| Birch | threshold=0.2 | 6 | 0.1899 |
| Birch | threshold=0.2 | 5 | 0.1780 |
| Birch | threshold=0.2 | 4 | 0.1775 |
| Birch | threshold=0.1 | 7 | 0.1709 |
| Birch | threshold=0.1 | 6 | 0.1649 |
| Birch | threshold=0.2 | 7 | 0.1599 |
| Birch | threshold=0.1 | 8 | 0.1542 |
| Birch | threshold=0.1 | 9 | 0.1530 |
| Birch | threshold=0.05 | 9 | 0.1395 |

**Supplementary Table 2.** Clustering algorithm optimization for the COVID cohort across 24 Birch hyperparameter configurations.

| **Model Type** | **Parameter Configuration** | **Clusters** | **Silhouette Score** |
| --- | --- | --- | --- |
| Birch | threshold=0.05 | 2 | 0.4254 |
| Birch | threshold=0.1 | 2 | 0.3934 |
| Birch | threshold=0.2 | 2 | 0.3830 |
| Birch | threshold=0.05 | 4 | 0.3317 |
| Birch | threshold=0.05 | 3 | 0.3297 |
| Birch | threshold=0.1 | 4 | 0.3176 |
| Birch | threshold=0.1 | 3 | 0.3134 |
| Birch | threshold=0.2 | 4 | 0.3078 |
| Birch | threshold=0.2 | 3 | 0.2939 |
| Birch | threshold=0.2 | 5 | 0.2902 |
| Birch | threshold=0.1 | 5 | 0.2443 |
| Birch | threshold=0.05 | 5 | 0.2379 |
| Birch | threshold=0.2 | 8 | 0.2369 |
| Birch | threshold=0.2 | 6 | 0.2347 |
| Birch | threshold=0.2 | 7 | 0.2345 |
| Birch | threshold=0.2 | 9 | 0.2249 |
| Birch | threshold=0.1 | 6 | 0.2045 |
| Birch | threshold=0.1 | 7 | 0.1995 |
| Birch | threshold=0.1 | 8 | 0.1965 |
| Birch | threshold=0.05 | 6 | 0.1884 |
| Birch | threshold=0.1 | 9 | 0.1814 |
| Birch | threshold=0.05 | 7 | 0.1755 |
| Birch | threshold=0.05 | 9 | 0.1591 |
| Birch | threshold=0.05 | 8 | 0.1573 |

**Supplementary Table 3.** Clustering algorithm optimization for the pre-COVID cohort across 56 hyperparameter configurations. Top 20 scoring configurations ordered by silhouette score.

| **Model Type** | **Parameter Configuration** | **Clusters** | **Silhouette Score** |
| --- | --- | --- | --- |
| KMeans | n_clusters=2 | 2 | 0.4666 |
| Birch | threshold=0.2 | 2 | 0.4572 |
| Birch | threshold=0.05 | 2 | 0.4413 |
| Birch | threshold=0.1 | 2 | 0.4346 |
| KMeans | n_clusters=3 | 3 | 0.3418 |
| Birch | threshold=0.1 | 3 | 0.3230 |
| Birch | threshold=0.05 | 3 | 0.2996 |
| Birch | threshold=0.1 | 4 | 0.2875 |
| Birch | threshold=0.05 | 5 | 0.2776 |
| Birch | threshold=0.05 | 4 | 0.2767 |
| KMeans | n_clusters=4 | 4 | 0.2718 |
| Birch | threshold=0.2 | 3 | 0.2582 |
| KMeans | n_clusters=6 | 6 | 0.2518 |
| KMeans | n_clusters=7 | 7 | 0.2476 |
| KMeans | n_clusters=5 | 5 | 0.2381 |
| KMeans | n_clusters=8 | 8 | 0.2367 |
| Birch | threshold=0.05 | 6 | 0.2246 |
| KMeans | n_clusters=9 | 9 | 0.2223 |
| DBSCAN | min_samples=13 | 4 | 0.2111 |
| Birch | threshold=0.2 | 9 | 0.2025 |

**Supplementary Table 4.** Clustering algorithm optimization for the COVID cohort across 56 hyperparameter configurations. Top 20 scoring configurations ordered by silhouette score.

| **Model Type** | **Parameter Configuration** | **Clusters** | **Silhouette Score** |
| --- | --- | --- | --- |
| KMeans | n_clusters=2 | 2 | 0.4602 |
| Birch | threshold=0.05 | 2 | 0.4254 |
| Birch | threshold=0.1 | 2 | 0.3934 |
| Birch | threshold=0.2 | 2 | 0.3830 |
| KMeans | n_clusters=4 | 4 | 0.3447 |
| KMeans | n_clusters=3 | 3 | 0.3399 |
| Birch | threshold=0.05 | 4 | 0.3317 |
| Birch | threshold=0.05 | 3 | 0.3297 |
| GaussianMixture | max_iter=1000, tol=0.0001 | 2 | 0.3182 |
| Birch | threshold=0.1 | 4 | 0.3176 |
| Birch | threshold=0.1 | 3 | 0.3134 |
| Birch | threshold=0.2 | 4 | 0.3078 |
| Birch | threshold=0.2 | 3 | 0.2939 |
| Birch | threshold=0.2 | 5 | 0.2902 |
| KMeans | n_clusters=5 | 5 | 0.2885 |
| KMeans | n_clusters=6 | 6 | 0.2537 |
| KMeans | n_clusters=7 | 7 | 0.2484 |
| Birch | threshold=0.1 | 5 | 0.2443 |
| DBSCAN | min_samples=13 | 4 | 0.2405 |
| DBSCAN | min_samples=16 | 4 | 0.2404 |

**Supplementary Figure 1.** Birch Silhouette analyses for 2 to 5 clusters on pre-COVID cohort.

**
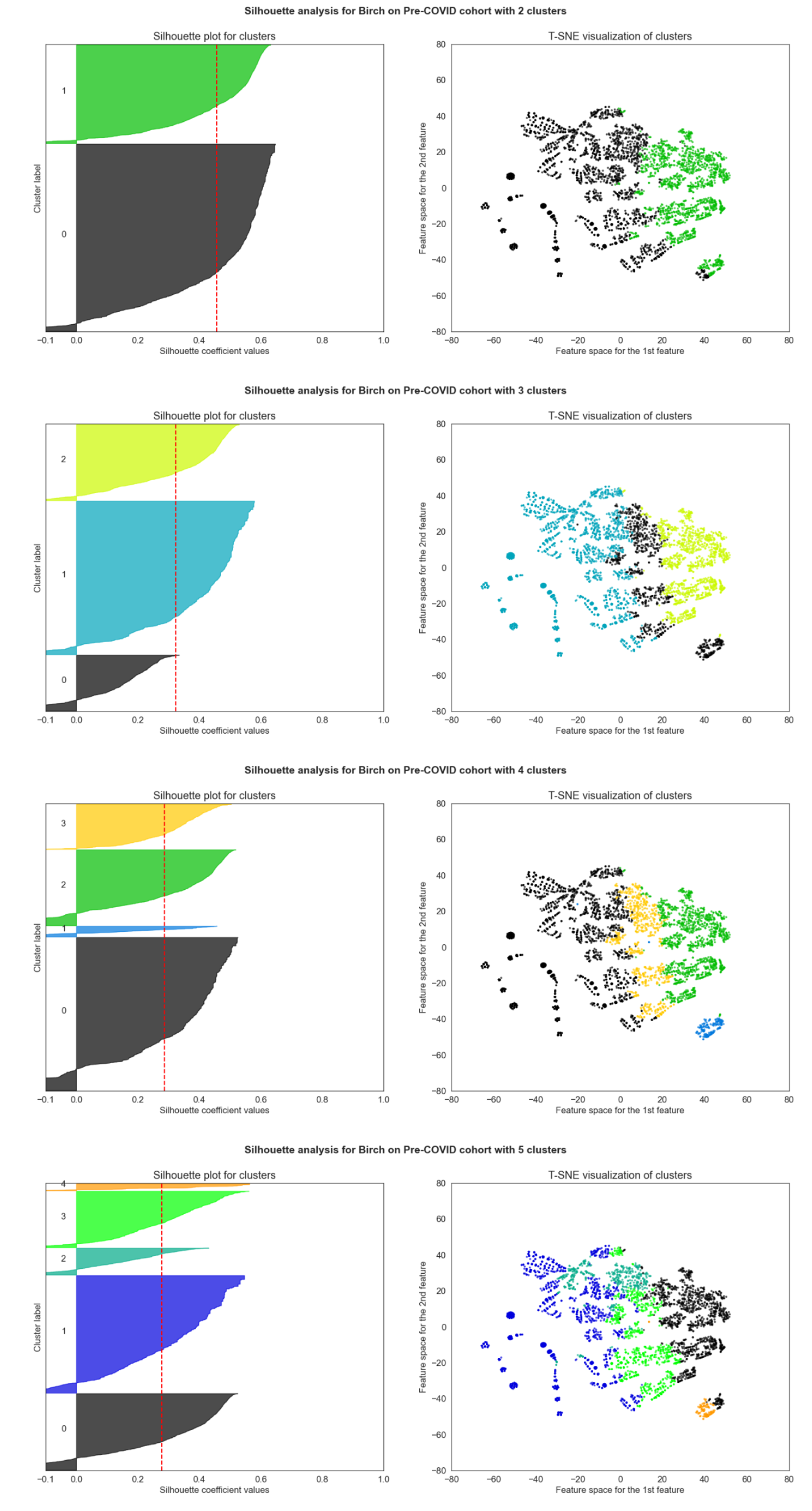
**

**Supplementary Figure 2.** Birch Silhouette analyses for 2 to 5 clusters on COVID cohort.


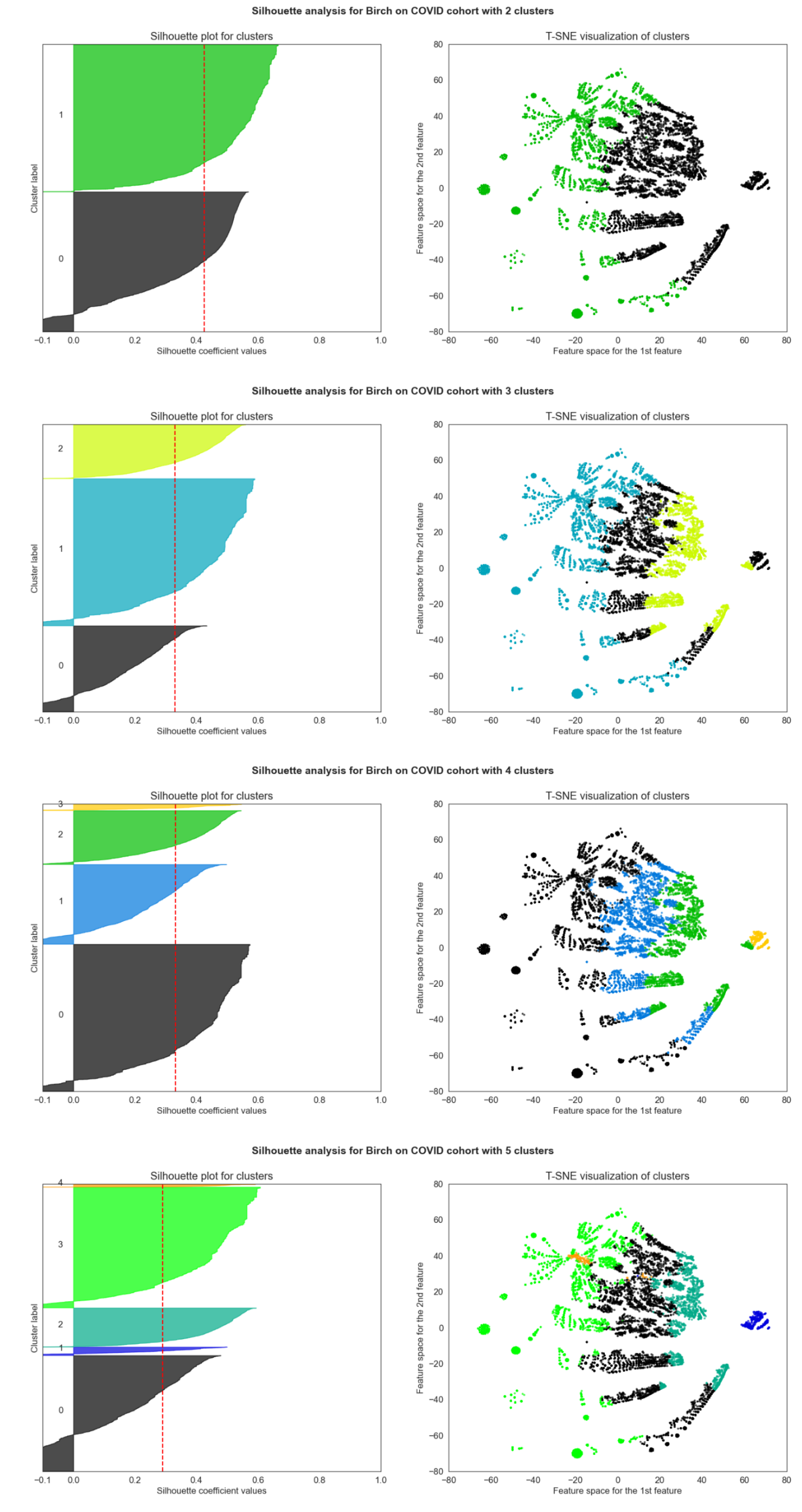


**Supplementary Figure 3.** KMeans Silhouette analyses for 2 to 5 clusters on pre-COVID cohort.

**
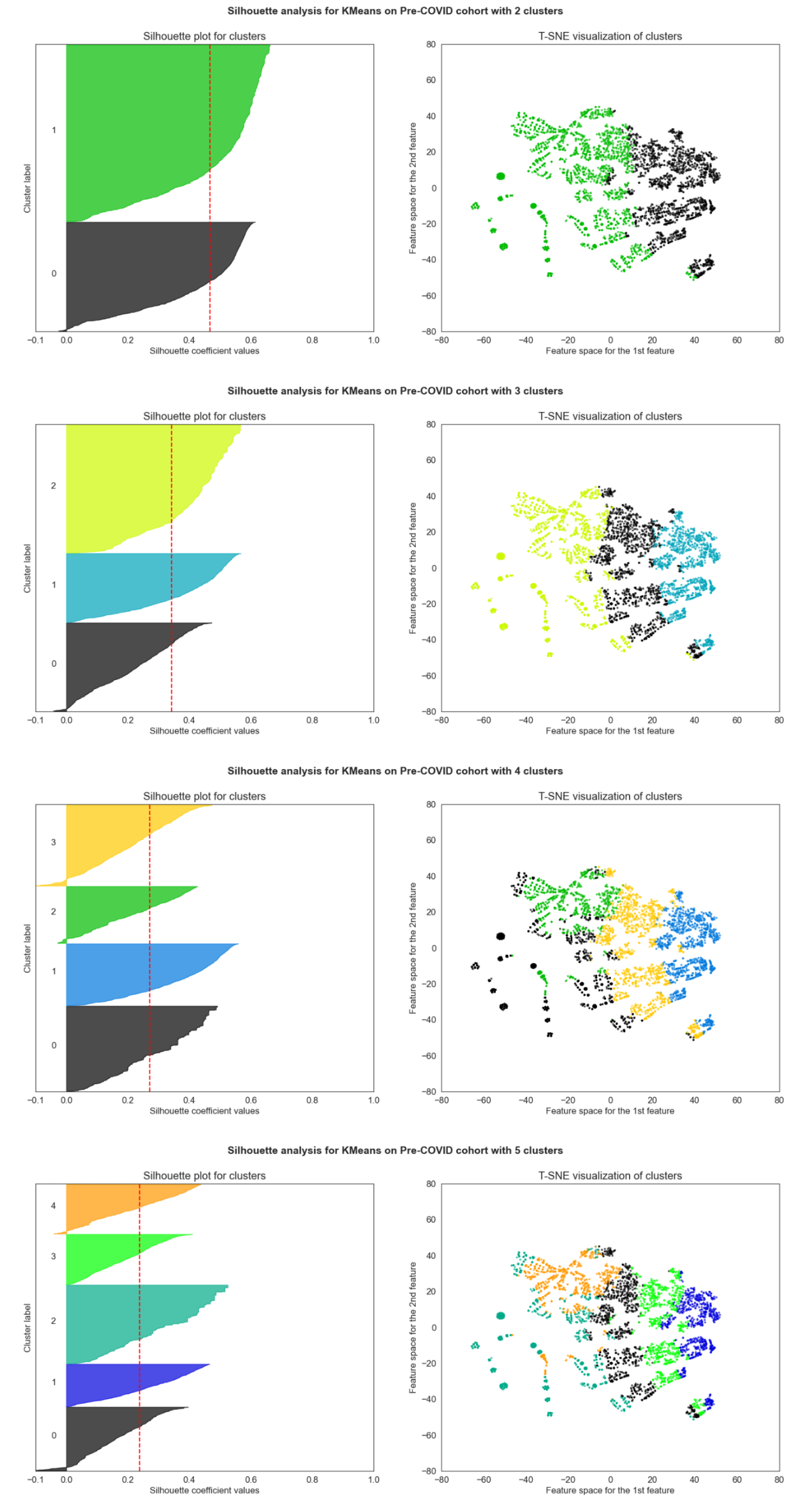
**

**Supplementary Figure 4.** KMeans Silhouette analyses for 2 to 5 clusters on COVID cohort.


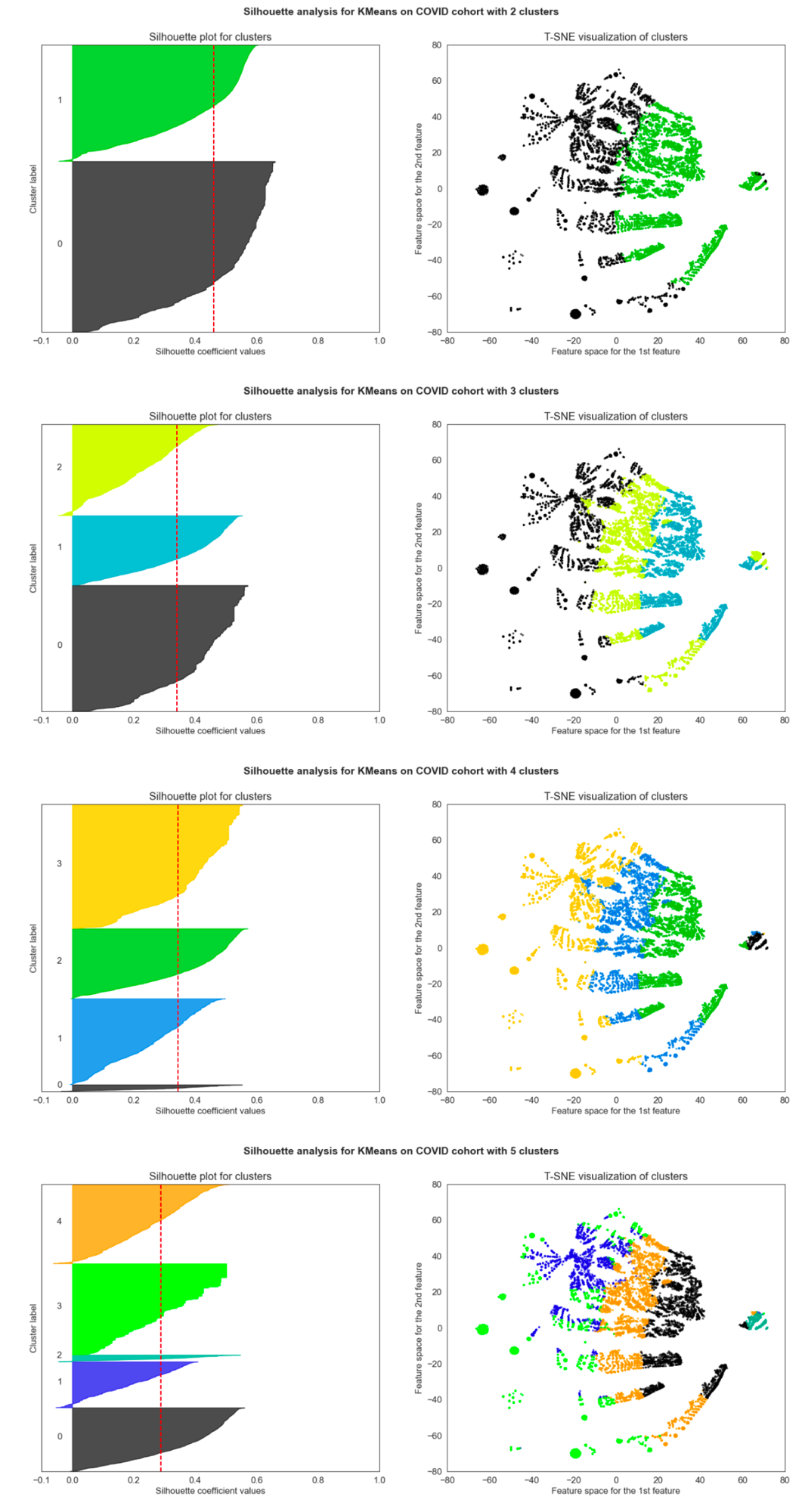


**Supplementary Figure 5.** Distribution plots for Period, Activeness and Engagement variables across pre-COVID and COVID KMeans clusters.


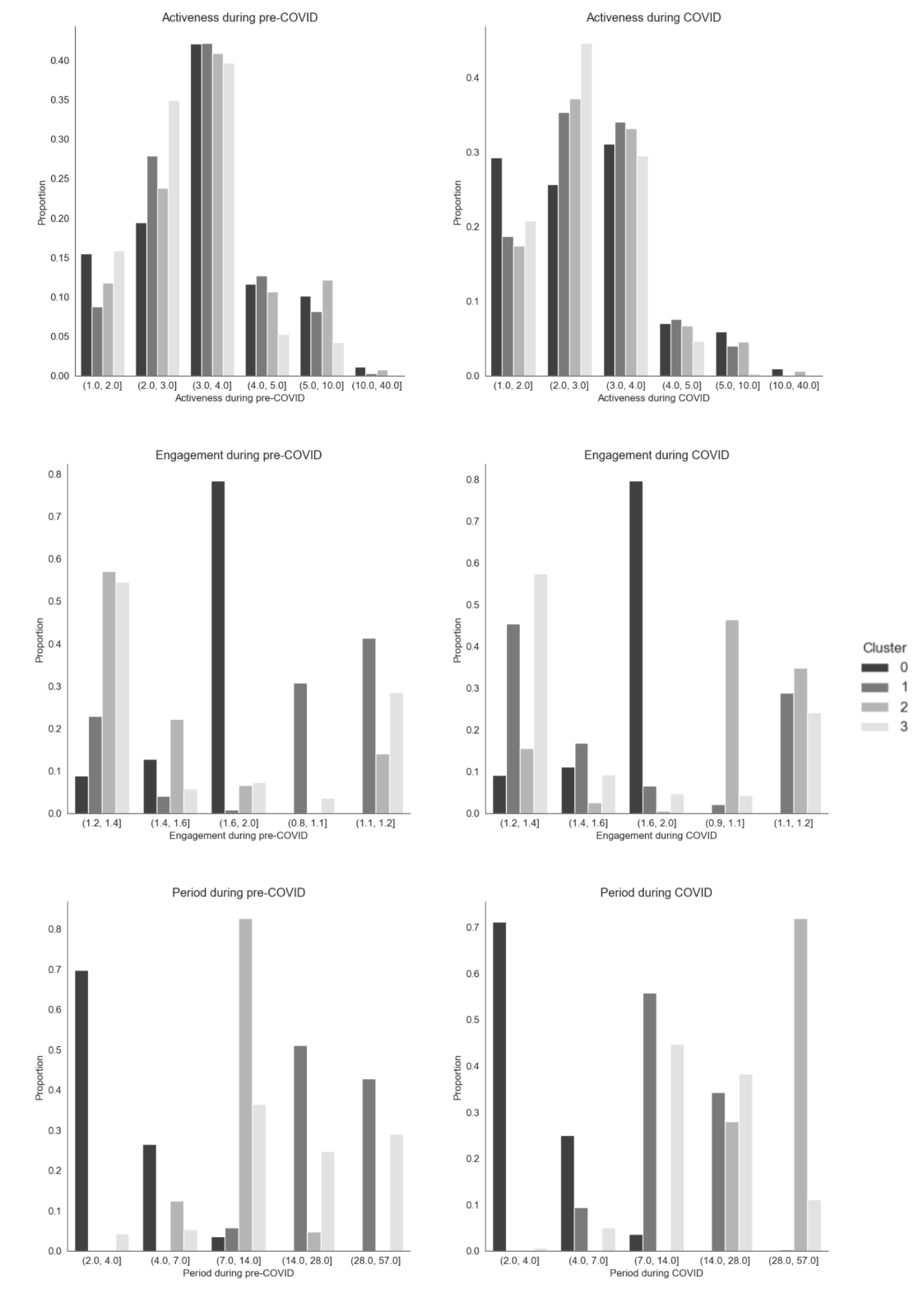


**Supplementary Table 5.** Variable distributions for pre-COVID and COVID cohorts. Adjusted p-value assesses whether each variable changed during COVID, compared to pre-COVID.

| Variable | Pre-COVID | COVID | Adjusted p-value |
| --- | --- | --- | --- |
| **Summary Statistics** | | | |
| Signups | 5048 | 13841 |  |
| Signups per day | 19.72 | 24.28 |  |
| **Demographic variables / Control variables not used for clustering** | | | |
| Gender |  |  |  |
| Female | 3899 (77.24% [76.06, 78.37]) | 10406 (75.18% [74.46, 75.89]) |  |
| Male | 971 (19.24% [18.17, 20.35]) | 2629 (18.99% [18.35, 19.66]) |  |
| Non-binary | 178 (3.53% [3.05, 4.07]) | 806 (5.82% [5.45, 6.23]) | <0.001 |
| Age group |  |  |  |
| 14-17 | 4683 (92.77% [92.02, 93.45]) | 12290 (88.79% [88.26, 89.31]) |  |
| 18-25 | 365 (7.23% [6.55, 7.98]) | 1551 (11.21% [10.69, 11.74]) | <0.001 |
| Ethnicity group |  |  |  |
| White | 4259 (84.37% [83.34, 85.35]) | 11280 (81.5% [80.84, 82.14]) |  |
| Asian | 331 (6.56% [5.91, 7.27]) | 917 (6.63% [6.22, 7.05]) |  |
| Black | 141 (2.79% [2.37, 3.28]) | 471 (3.4% [3.11, 3.72]) |  |
| Mixed | 239 (4.73% [4.18, 5.36]) | 727 (5.25% [4.89, 5.64]) |  |
| Other | 78 (1.55% [1.24, 1.92]) | 446 (3.22% [2.94, 3.53]) | <0.001 |
| **Service usage variables / Dependent variables used for clustering** | | | |
| Period |  | - | 0.016 |
| Engagement |  | - | 0.074 |
| Activeness |  | - | <0.001 |
| Journal entry | 3716 (73.61% [72.38, 74.81]) | 11675 (84.35% [83.74, 84.95]) | <0.001 |
| Personal goal created | 1007 (19.95% [18.87, 21.07]) | 2418 (17.47% [16.85, 18.11]) | <0.001 |
| Article created | 296 (5.86% [5.25, 6.55]) | 466 (3.37% [3.08, 3.68]) | <0.001 |
| Discussion created | 1051 (20.82% [19.72, 21.96]) | 2741 (19.8% [19.15, 20.48]) | 0.251 |
| Comment created | 1644 (32.57% [31.29, 33.87]) | 4772 (34.48% [33.69, 35.27]) | 0.008 |
| Message sent | 771 (15.27% [14.31, 16.29]) | 2237 (16.16% [15.56, 16.78]) | 0.129 |
| Ad-hoc chat | 3791 (75.1% [73.89, 76.27]) | 7816 (56.47% [55.64, 57.29]) | <0.001 |
| Booked chat | 196 (3.88% [3.38, 4.45]) | 403 (2.91% [2.64, 3.21]) | 0.001 |
| **Service experience variables / Observational variables not used for clustering** | | | |
| Administrative message received | 475 (9.41% [8.63, 10.25]) | 7553 (54.57% [53.74, 55.4]) | <0.0001 |
| Therapeutic message received | 680 (13.47% [12.56, 14.44]) | 7152 (51.67% [50.84, 52.5]) | <0.0001 |
| Successful chat | 1567 (31.04% [29.78, 32.33]) | 2611 (18.86% [18.22, 19.52]) | <0.0001 |
| Failed chat | 1065 (21.1% [19.99, 22.24]) | 2675 (19.33% [18.68, 19.99]) | 0.008 |

**Supplementary Table 6 (Part 1).** Control variables across KMeans engagement clusters for the pre-COVID cohort. Reported values are absolute counts and percentages with 95% confidence intervals. Chi-squared tests across clusters returned p<0.001 for all variables except otherwise specified. In red and blue, standing out highest and lowest values across clusters respectively.

| Variable | C0 | C1 | C2 | C3 |
| --- | --- | --- | --- | --- |
| **Pre-COVID: Summary Statistics** | | | | |
| **Signups** | **1510** | **1434** | **1095** | **1009** |
| Proportion of all pre-COVID signups | 29.91% | 28.41% | 21.69% | 19.99% |
| Signups per day | 5.90 | 5.60 | 4.28 | 3.94 |
| **Pre-COVID: Control variables not used for clustering** | | | | |
| Gender (p=0.547) | | | | |
| Female | 1174 (77.75% [75.58, 79.77]) | 1101 (76.78% [74.52, 78.89]) | 857 (78.26% [75.73, 80.61]) | 767 (76.02% [73.29, 78.55]) |
| Male | 295 (19.54% [17.61, 21.61]) | 279 (19.46% [17.49, 21.59]) | 193 (17.63% [15.48, 19.99]) | 204 (20.22% [17.85, 22.81]) |
| Non-binary | 41 (2.72% [2.01, 3.66]) | 54 (3.77% [2.9, 4.88]) | 45 (4.11% [3.09, 5.45]) | 38 (3.77% [2.76, 5.13]) |
| Age group | | | | |
| 14-17 | 1380 (91.39% [89.87, 92.7]) | 1332 (92.89% [91.44, 94.11]) | 1007 (91.96% [90.2, 93.43]) | 964 (95.54% [94.08, 96.65]) |
| 18-25 | 130 (8.61% [7.3, 10.13]) | 102 (7.11% [5.89, 8.56]) | 88 (8.04% [6.57, 9.8]) | 45 (4.46% [3.35, 5.92]) |
| Ethnicity group (p=0.042) | | | | |
| Asian | 104 (6.89% [5.72, 8.28]) | 75 (5.23% [4.19, 6.51]) | 89 (8.13% [6.65, 9.9]) | 63 (6.24% [4.91, 7.91]) |
| Black | 36 (2.38% [1.73, 3.28]) | 41 (2.86% [2.11, 3.86]) | 30 (2.74% [1.93, 3.88]) | 34 (3.37% [2.42, 4.67]) |
| Mixed | 69 (4.57% [3.63, 5.74]) | 64 (4.46% [3.51, 5.66]) | 48 (4.38% [3.32, 5.76]) | 58 (5.75% [4.47, 7.36]) |
| Other | 21 (1.39% [0.91, 2.12]) | 22 (1.53% [1.02, 2.31]) | 23 (2.1% [1.4, 3.13]) | 12 (1.19% [0.68, 2.07]) |
| White | 1280 (84.77% [82.87, 86.49]) | 1232 (85.91% [84.02, 87.62]) | 905 (82.65% [80.29, 84.78]) | 842 (83.45% [81.03, 85.61]) |
| Messages received | | | | |
| Message received | 219 (14.5% [12.82, 16.37]) | 293 (20.43% [18.43, 22.6]) | 322 (29.41% [26.78, 32.17]) | 192 (19.03% [16.73, 21.57]) |

**Supplementary Table 6 (Part 3).** Usage variables across KMeans engagement clusters for the pre-COVID cohort. Reported values are absolute counts and percentages with 95% confidence intervals. Logistic regression models adjusted for demographic variables returned p<0.001 for all variables unless otherwise specified. In red and blue, standing out highest and lowest values across clusters respectively.

| Variable | C0 | C1 | C2 | C3 |
| --- | --- | --- | --- | --- |
| **Signups** | **1510** | **1434** | **1095** | **1009** |
| **Pre-COVID: Dependent variables used for clustering** | | | | |
| Journal entry | 1042 (69.01% [66.63, 71.29]) | 1011 (70.5% [68.09, 72.81]) | 801 (73.15% [70.45, 75.69]) | 862 (85.43% [83.12, 87.47]) |
| Personal goal created | 155 (10.26% [8.83, 11.9]) | 271 (18.9% [16.96, 21.01]) | 243 (22.19% [19.83, 24.75]) | 338 (33.5% [30.65, 36.47]) |
| Article created | 24 (1.59% [1.07, 2.35]) | 85 (5.93% [4.82, 7.27]) | 75 (6.85% [5.5, 8.5]) | 112 (11.1% [9.31, 13.19]) |
| Discussion created | 89 (5.89% [4.81, 7.2]) | 285 (19.87% [17.89, 22.02]) | 256 (23.38% [20.97, 25.98]) | 421 (41.72% [38.72, 44.79]) |
| Comment created | 189 (12.52% [10.94, 14.28]) | 450 (31.38% [29.03, 33.83]) | 410 (37.44% [34.62, 40.35]) | 595 (58.97% [55.91, 61.96]) |
| Message sent | 116 (7.68% [6.44, 9.14]) | 242 (16.88% [15.03, 18.9]) | 225 (20.55% [18.26, 23.04]) | 188 (18.63% [16.35, 21.15]) |
| Ad-hoc chat | 1030 (68.21% [65.82, 70.51]) | 1126 (78.52% [76.32, 80.57]) | 889 (81.19% [78.76, 83.39]) | 746 (73.93% [71.14, 76.55]) |
| Booked chat | 10 (0.66% [0.36, 1.21]) | 99 (6.9% [5.7, 8.33]) | 84 (7.67% [6.24, 9.4]) | 3 (0.3% [0.1, 0.87]) |
| **Pre-COVID: Observational variables not used for clustering** | | | | |
| Successful chat | 287 (19.01% [17.11, 21.06]) | 562 (39.19% [36.7, 41.74]) | 469 (42.83% [39.93, 45.78]) | 249 (24.68% [22.12, 27.43]) |
| Failed chat | 223 (14.77% [13.07, 16.65]) | 363 (25.31% [23.13, 27.63]) | 298 (27.21% [24.66, 29.93]) | 181 (17.94% [15.69, 20.43]) |

**Supplementary Table 7 (Part 1).** Control variables across KMeans engagement clusters for the COVID cohort. Reported values are absolute counts and percentages with 95% confidence intervals. Chi-squared tests across clusters returned p<0.001 for all variables except otherwise specified. In red and blue, standing out highest and lowest values across clusters respectively.

| Variable | C0 | C1 | C2 | C3 |
| --- | --- | --- | --- | --- |
| **COVID: Summary Statistics** | | | | |
| **Signups** | **5989** | **4151** | **3380** | **321** |
| Proportion of all COVID signups | 43.26% | 29.99% | 24.42% | 2.32% |
| Signups per day | 10.51 | 7.28 | 5.93 | 0.56 |
| **COVID: Control variables (not used for clustering)** | | | | |
| Gender | | | | |
| Female | 4416 (73.74% [72.61, 74.83]) | 3101 (74.7% [73.36, 76.0]) | 2629 (77.78% [76.35, 79.15]) | 260 (81.0% [76.35, 84.91]) |
| Male | 1216 (20.3% [19.3, 21.34]) | 802 (19.32% [18.15, 20.55]) | 559 (16.54% [15.32, 17.83]) | 52 (16.2% [12.57, 20.63]) |
| Non-binary | 357 (5.96% [5.39, 6.59]) | 248 (5.97% [5.29, 6.74]) | 192 (5.68% [4.95, 6.51]) | 9 (2.8% [1.48, 5.24]) |
| Age group (p=0.005) | | | | |
| 14-17 | 5317 (88.78% [87.96, 89.55]) | 3702 (89.18% [88.2, 90.09]) | 3009 (89.02% [87.93, 90.03]) | 262 (81.62% [77.02, 85.47]) |
| 18-25 | 672 (11.22% [10.45, 12.04]) | 449 (10.82% [9.91, 11.8]) | 371 (10.98% [9.97, 12.07]) | 59 (18.38% [14.53, 22.98]) |
| Ethnicity group (p=0.011) | | | | |
| Asian | 359 (5.99% [5.42, 6.62]) | 290 (6.99% [6.25, 7.8]) | 254 (7.51% [6.67, 8.45]) | 14 (4.36% [2.62, 7.19]) |
| Black | 217 (3.62% [3.18, 4.13]) | 145 (3.49% [2.98, 4.1]) | 102 (3.02% [2.49, 3.65]) | 7 (2.18% [1.06, 4.43]) |
| Mixed | 315 (5.26% [4.72, 5.85]) | 205 (4.94% [4.32, 5.64]) | 194 (5.74% [5.0, 6.58]) | 13 (4.05% [2.38, 6.8]) |
| Other | 212 (3.54% [3.1, 4.04]) | 130 (3.13% [2.64, 3.71]) | 93 (2.75% [2.25, 3.36]) | 11 (3.43% [1.92, 6.03]) |
| White | 4886 (81.58% [80.58, 82.54]) | 3381 (81.45% [80.24, 82.6]) | 2737 (80.98% [79.62, 82.26]) | 276 (85.98% [81.76, 89.36]) |
| Messages received | | | | |
| Message received | 4472 (74.67% [73.55, 75.76]) | 3141 (75.67% [74.34, 76.95]) | 2751 (81.39% [80.04, 82.67]) | 295 (91.9% [88.4, 94.41]) |

**Supplementary Table 7 (Part 2).** Usage variables across KMeans engagement clusters for the COVID cohort. Reported values are absolute counts and percentages with 95% confidence intervals. Logistic regression models adjusted for demographic variables returned p<0.001 for all variables unless otherwise specified. In red and blue, standing out highest and lowest values across clusters respectively.

| Variable | C0 | C1 | C2 | C3 |
| --- | --- | --- | --- | --- |
| **Signups** | **5989** | **4151** | **3380** | **321** |
| **COVID: Dependent variables used for clustering** | | | | |
| Journal entry | 5125 (85.57% [84.66, 86.44]) | 3517 (84.73% [83.6, 85.79]) | 2875 (85.06% [83.82, 86.22]) | 158 (49.22% [43.79, 54.67]) |
| Personal goal created | 947 (15.81% [14.91, 16.76]) | 758 (18.26% [17.11, 19.47]) | 701 (20.74% [19.41, 22.14]) | 12 (3.74% [2.15, 6.42]) |
| Article created | 164 (2.74% [2.35, 3.18]) | 143 (3.44% [2.93, 4.04]) | 157 (4.64% [3.99, 5.41]) | 2 (0.62% [0.17, 2.24]) |
| Discussion created | 1068 (17.83% [16.88, 18.82]) | 827 (19.92% [18.74, 21.17]) | 838 (24.79% [23.37, 26.28]) | 8 (2.49% [1.27, 4.84]) |
| Comment created | 1923 (32.11% [30.94, 33.3]) | 1442 (34.74% [33.3, 36.2]) | 1355 (40.09% [38.45, 41.75]) | 52 (16.2% [12.57, 20.63]) |
| Message sent | 726 (12.12% [11.32, 12.97]) | 685 (16.5% [15.4, 17.66]) | 742 (21.95% [20.59, 23.38]) | 84 (26.17% [21.66, 31.24]) |
| Ad-hoc chat | 3080 (51.43% [50.16, 52.69]) | 2338 (56.32% [54.81, 57.83]) | 2119 (62.69% [61.05, 64.31]) | 279 (86.92% [82.79, 90.17]) |
| Booked chat | 3 (0.05% [0.02, 0.15]) | 21 (0.51% [0.33, 0.77]) | 58 (1.72% [1.33, 2.21]) | 321 (100.0% [98.82, 100.0]) |
| **COVID: Observational variables not used for clustering** | | | | |
| Successful chat | 676 (11.29% [10.51, 12.11]) | 779 (18.77% [17.61, 19.98]) | 861 (25.47% [24.03, 26.97]) | 295 (91.9% [88.4, 94.41]) |
| Failed chat | 922 (15.39% [14.5, 16.33]) | 768 (18.5% [17.35, 19.71]) | 727 (21.51% [20.16, 22.93]) | 258 (80.37% [75.68, 84.35]) |
